# Efficacy of mobile phone intervention to increase male partner antenatal care attendance for HIV testing in Moshi municipal, Tanzania: a randomized controlled trial

**DOI:** 10.1186/s12884-024-06337-x

**Published:** 2024-04-24

**Authors:** Angela Lyimo, Blandina Mmbaga, Ashraf Mahmoud, Wilson Saimon Eliamini, Nicolaus Bartholomew Ngowi, Modesta Mitao, Godwin Pancras, Evangelista Malindisa, Paulo Kidayi, Donaldson F. Conserve, John Bartlett, Bruno Sunguya, Eligius Lyamuya, Benson Kidenya, Emmanuel Balandya, James Samwel Ngocho

**Affiliations:** 1 Faculty of Nursing, Kilimanjaro Christian Medical University College, Box 2240, Moshi, Tanzania; 2 Kilimanjaro Clinical Research Institute, Box 2236, Moshi, Tanzania; 3 Faculty of Medicine, Kilimanjaro Christian Medical University College, Box 2240, Moshi, Tanzania; 4 Kilimanjaro Christian Medical Centre, Box 3010, Moshi, Tanzania; 5 Department of Epidemiology and Applied Biostatistics, Institute of Public Health, Kilimanjaro Christian Medical University College, Box 2240, Moshi, Tanzania; 6 Catholic University of Health and Allied Science, Box 1464, Mwanza, Tanzania; 7 Muhimbili University of Health and Allied Science, Box 65001, Dar es Salam, Tanzania; 8 Milken Institute of Public Health, The George Washington University, Washington, DC USA; 9 Duke Global Health Institute, Durham, NC USA

**Keywords:** Antenatal care, Prevention of mother-to-child transmission, HIV, Male engagement, Maternal health, Partner support, Tanzania

## Abstract

**Background:**

HIV partner counselling and testing in antenatal care (ANC) is a crucial strategy to raise the number of males who know their HIV status. However, in many settings like Tanzania, male involvement in antenatal care remains low, and there is a definite need for innovative strategies to increase male partner involvement. This study was designed to evaluate the efficacy of mobile phone intervention increase male partner ANC attendance for HIV testing in Moshi municipal, Tanzania.

**Methods:**

Between April and July 2022, we enrolled pregnant women presenting to a first ANC visit at Majengo and St. Joseph reproductive health facilities without their male partners. Eligible pregnant women were randomly assigned to invitation of their male partners either via phone calls, text messages from clinic staff and verbal invites from pregnant partners (intervention arm) or verbal invites only from the pregnant partners (control arm). Neither healthcare provider nor participant were blinded. The primary outcome was the proportion of male partners who attended ANC with their pregnant partners during a follow-up period of two consecutive visits. The secondary outcome measure was HIV testing among male partners following the invitation. Participants were analyzed as originally assigned (intention to treat).

**Results:**

A total of 350 pregnant women presenting to ANC for the first time were enrolled, with 175 women enrolled in each arm. The efficacy of male attendance with their pregnant women following the invitations was 83.4% (147/175) in the intervention arm and 46.3% (81/175) in the control arm. Overall, the results suggest a positive and statistically significant average treatment effect among men who received mobile phone intervention on ANC attendance. For the secondary outcome, the percent of male partners who accepted HIV counselling and testing was 99.3% (146/147) in the intervention arm and 93.8% (76/81) in the control arm. Married men were having higher odds of ANC attendance compared with single men (aOR:6.40(3.26–12.56), Males with multigravida women were having lower odds of ANC attendance compared with primigravida women (aOR:0.17(0.09–0.33).

**Conclusion:**

The study demonstrates that supplementing verbal invitations with mobile phone calls and text messages from clinic staff can significantly increase male partner ANC attendance and HIV testing. This combined approach is recommended in improving ANC attendance and HIV testing of male partners who do not accompany their pregnant partners to antenatal clinics in the first visits.

**Trial registration:**

PACTR202209769991162.

## Background

Human immunodeficiency virus (HIV) affects approximately 38.4 million people worldwide, with 650,000 HIV-related deaths occurring each year [1]. Though the burden of HIV varies significantly over the globe, Africa is more severely affected accounting for 71% of HIV/AIDS-related deaths [2] In Tanzania the prevalence of HIV estimated to be 3.8% among adult aged 15–49 years with (5.0% in women and 2.4% in men [3–5]. The low prevalence in men is probably due to under-diagnosis, as it has been reported that about 55% of men living with HIV do not know their status [5]. Surprisingly, more than half of all deaths related to HIV infections occur in men [5]. The prevalence of HIV among pregnant women attending their first ANC visit was found to be 6.3% in 2021, and the majority of their male partners were unaware of this [6].

Following WHO recommendations, the Prevention of Mother-to-Child Transmission (PMTCT) of HIV program has been included in Antenatal Care (ANC) to increase partner HIV counselling and testing in different parts of the world [7]. The implementation of PMTCT services has played a crucial role in preventing approximately 1.4 million HIV infections among children from 2010 to 2018 [8]. PMTCT testing significantly lowers the risk of HIV transmission from an HIV-positive mother to her baby during pregnancy, labor, delivery, or breastfeeding [9].

HIV partner counselling and testing in ANC is a crucial strategy to raise the number of males who know their HIV status. Currently, to achieve this, a male invitation to ANC is used and remains the responsibility of a pregnant woman [10]. For over ten years, Tanzania has engaged male partners of pregnant women in ANC through routine HIV counselling and testing [10, 11] as part of the prevention of mother to child transmission (PMTCT) program. Along with this recommendation, pregnant women are usually required to invite their partners during their first ANC visits to receive all the necessary information and services for the pregnancy, including HIV counselling and testing [12].

Regardless of this approach, the response seems to be low due to several factors, including domestic violence, infidelity, and fear of HIV-positive results, which make pregnant women less likely to invite their male partners [13]. Alternative strategies such as women who come with their partners being given priority, service rejection to those without their partners [14], and continuous community sensitization have been tried, but the response is still low, with only 30% of men attending ANC with their partners [15].On the other hand, more than 90% of pregnant women attending ANC get HIV counselling and testing while only 8% of men test for HIV during ANC [15].

Increased access to mobile phones and expansion to mobile network and its innovations in mobile technologies offers an opportunity to utilize mobile services for public health benefit. The use of mobile phones has been proven to help overcome some of the health care challenges in different parts of the world. In Tanzania 83% of its people have access to mobile phone and access to mobile network which enable people to communicate and share different information [16].Different strategies need to be applied to increase number of males who knows their HIV status. This has also proven in Tanzania toward the strategies to reduce transmission of HIV pandemic on the use of Pre-Exposure Prophylaxis(PrEP) to the group exposed to the mobile phones the retention was higher compared to the other group [17].Failure of male partner attendance in ANC has been identified as a barrier to successful PMTCT measures and efforts to achieve the 95, 95, 95 policy goal [5]. This interventional study evaluated the efficacy of mobile phone intervention to increase male partner antenatal care attendance for HIV testing in Moshi Municipal, Tanzania.

## Methods

### Study design and setting

This was a randomized controlled trial study design with two arms. The intervention arm comprised of mobile phone intervention (calls and text messages) from healthcare provider plus verbal invitations from pregnant partner to her male partner for the ANC invitation. The study was conducted in Moshi Municipality in Kilimanjaro region. The municipality has population of 221,733 [18].The municipality has 4 hospitals, 11 health centers, 35 Dispensaries, and 7 clinics. The study was carried at Majengo health center and St. Joseph designated district hospital reproductive health clinics in Moshi municipality from April 2022 to July 2022. The two reproductive health facilities serve large population in the municipality with approximately 2500 pregnant women per year; an estimated 4.8% of pregnant women seen at the clinics are living with HIV [19]. Pregnant women are encouraged to bring male partner to the first ANC visit for pregnancy education as well as partner HIV testing but local data shows low response.

### Study population

Women aged 18–49 years who attended ANC for the first time during their current pregnancy were eligible for inclusion. The study followed these women for two months, inviting them to bring their partners after their initial ANC visit. Eligibility criteria encompassed women with a confirmed pregnancy according to the health facility’s findings. HIV-positive mothers whose partners were unaware of their HIV status were included, while those whose partners were already aware and linked to ART and treatment services were excluded. Additionally, critically ill partners and widows were excluded from the study.

### Study variables

#### Primary outcome variables

Male partner attendance: Defined as the number of male partners who attended with their pregnant partners to the ANC within 8 weeks (two months) following the invitation from the first ANC visit.

#### Secondary outcome variables

HIV counseling and testing: Defined as the number of male partners who attended ANC following the invitation and accepted HIV counseling and testing.

#### Exposure variables

Social demographic information including maternal age, marital status, education, employment, pregnancy type, HIV status, History of pregnancy complication, and Gravidity.

### Sample size and power

Sample size calculation was based on the primary outcome. Our estimate assumed 0.52 reported to attend in for Control group and 0.74 in intervention [20]. On the basis of a 1:1 allocation ratio, a two-sided α of 0.05 and 80% power used to estimate the sample size.

$$n = \left[ {{{\left( {{Z_{\alpha /2}} + {Z_\beta }} \right)}^2} \times \left\{ {(p1\left( {1 - p1} \right) + \left( {p2\left( {1 - p2} \right)} \right)} \right\}} \right]/{\left( {p1 - p2} \right)^2}$$

where.

n = sample size required in each group,

p1 = proportion of male partners attended in intervention arm = 0.74.

p2 = proportion of male partners attended in control group arm = 0.52.

p1-p2 = 0.22.

Z_α/2_: This depends on level of significance, for 5% this is 1.96.

Z_β_: This depends on power, for 80% this is 0.84.

From the formula the sample size required per group 175.The total sample size required is 350.

Based on a 1:1 allocation ratio, a two-sided α of 0.05 and 80% power we estimated a sample size of 350 each study arm with 175.participants.

### Study procedures

**Fig. 1:**
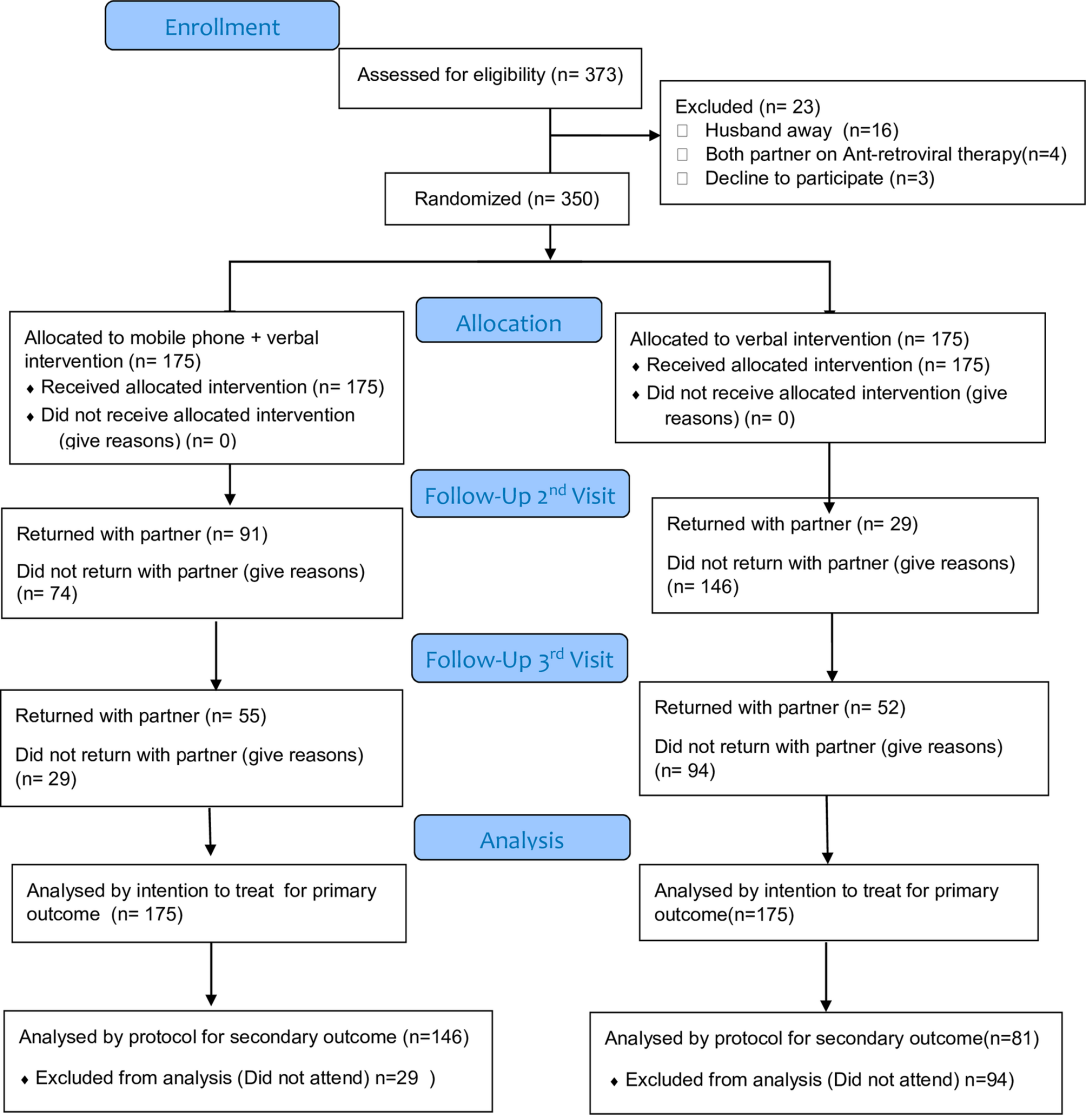
Flowchart of study participants enrollment procedures

A total of 373 pregnant women who attended their first ANC visit were assessed for the eligibility criteria by the research assistant, of which 350 were enrolled and randomly assigned to either intervention arm or control arm (175 women in each group) as illustrated in Fig. 1.

### Interventional

**Fig. 2:**
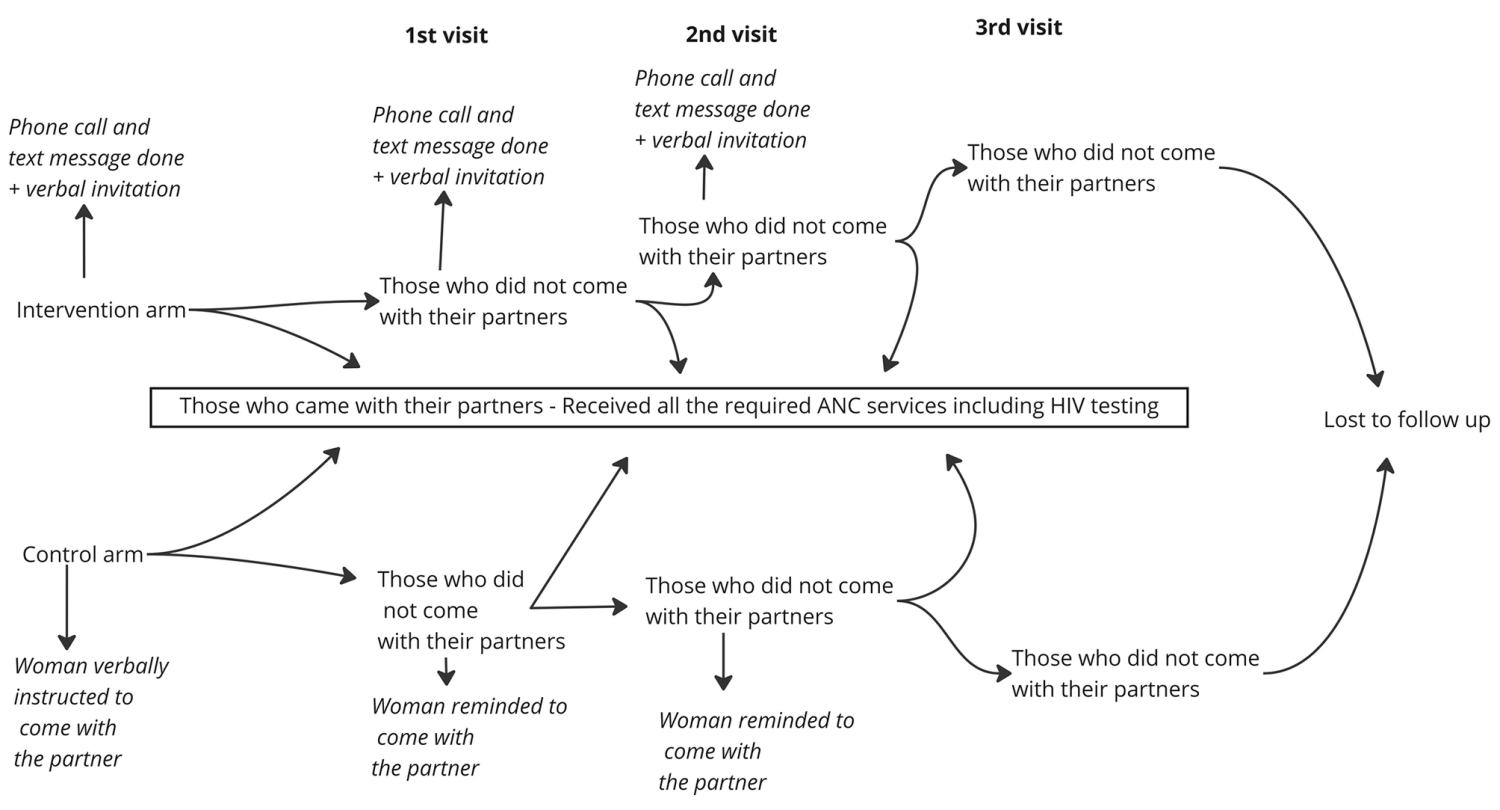
A figure describing the Intervention and Control arm follow-up

The intervention comprised mobile phone calls and text messages from healthcare providers, complemented by verbal invitations from pregnant partners to their male counterparts for ANC attendance. Study participants were recruited by the researcher and research assistant, with eligible individuals enrolled when pregnant women first arrived at the ANC clinic, before receiving their initial ANC service. Upon assignment, participants were directed to routine healthcare services, in addition to the intervention allocated by the research assistant. Therefore, the intervention was administered on the same day as recruitment. Healthcare providers used calls and text messages to inform male partners about the ANC invitation. Subsequently, male partners were reminded of their ANC appointment weekly through the same communication channels until they attended ANC with their pregnant partners. The first call and message were initiated on the day of allocation, following the receipt of the first ANC services. Healthcare providers maintained weekly communication with male partners to reinforce the ANC invitation. A maximum of eight mobile phone calls and eight text messages were administered. Male partners who did not attend the ANC clinic following the maximum communication attempts were considered non-respondents. All communication was conducted in Swahili. The follow-up period for visits extended to two months (8 weeks). Healthcare providers and study participants were not blinded (**see** Fig. 2).

### Control

In the control group, each pregnant woman verbally invited her male partner to accompany her on the next ANC visit, as illustrated in Fig. 2. Pregnant women who came without their male partners to the second visit were reminded to continue inviting their male partners to the next visit. Pregnant women who did not come with their partners within 8 weeks during the second and third visits were considered non-respondents.

### Randomization and blinding

Simple randomization was used and the unit of randomization was pregnant woman who were randomly allocated to one of two, either the intervention arm (mobile phone plus verbal invitation) or control (verbal invitation only). The same day of the first ANC visit research assistants, randomly assigned the participants into two parallel groups - the intervention and control arms with an allocation ratio of 1:1. An investigator was responsible for computerized sequence generation program on allocation in two groups (intervention and control arms), where the number “0” generated by the program was considered as “control” arm and number “1” considered as “intervention” arm. Lists of numbers were generated separately for each of the two study health facilities. The research assistant did allocation concealment. Research assistant conduct enrolment to the participants who meeting inclusion criteria, consenting the participants, and completing baseline assessments participants given number which related to the group assignment. The participant presents the number to the healthcare provider who will also provide the first ANC services opened corresponding study identity and give the intervention. The healthcare provider and study participants were not blinded to study group assignment because the intervention required overt participation.

### Statistical analysis

Data cleaning and analysis was done using Stata version 15.0 (Stata Corp, College Station, TX). Descriptive statistics were summarized using median with their respective interquartile range for continuous variables, and categorical variables were summarized using frequency and proportions presented in tables. A Chi-squared test was used to compare the proportion of male partners in attendance at the ANC following the invitation between the intervention and control arms by socio-demographic characteristics. A multivariable logistic regression model was used to determine maternal factors associated with male partner ANC attendance. In the multivariable model, we performed a priori selection of variables based on literature and included those with a significance level of *p* < 0.05 in the univariable analysis in the final multivariable model. Adjusted odds ratios (aOR) with their respective 95% confidence intervals were reported to show the magnitude of the association. To evaluate the efficacy of the mobile phone intervention, the average treatment effect (ATE) was used in this study. *P*-value < 0.05 was considered statistically significant.

## Results

### Baseline characteristics of pregnant women who attended first ANC visits in Moshi municipality

The baseline characteristics of pregnant women attending their first ANC visits in Moshi municipality were examined across control and intervention groups. In terms of socio-demographic features, both groups showed comparable maternal age distributions, with the majority 84.3% (295/350) falling in the 18–34 age range. Urban residence was predominant in both groups, and while a significant difference in marital status was noted (*p* = 0.013), the majority of the study participants had a higher proportion of 75.4% (264/350) for married or cohabiting participants.

Statistically significant differences in education levels were observed (*p* = 0.042. Employment status, health facility distribution, gravidity, pregnancy type, and HIV status showed no significant differences between the groups. Notably, a significant difference emerged in the history of complications in the previous pregnancy (*p* = 0.027), with a lower proportion of 10.9% (38/350) among study participants reporting complications as summarized in Table 1.

**Table 1:** Baseline characteristics of pregnant women who attended first ANC visits in Moshi municipality (*N* = 350)

Characteristics	Control	Intervention	Total	*P*-value
	(*n* = 175)	(*n* = 175)		
**Socio-demographic**				
**Maternal Age (in years)**				
18–34	141 (80.6)	154 (88.0)	295 (84.3)	0.056
35–49	34 (19.4)	21 (12.0)	55 (15.7)	
*Median (IQR)*			26(23,31)	
**Residence**				
Rural	44 (25.1)	54 (30.9)	98 (28.0)	0.234
Urban	131 (74.9)	121 (69.1)	252 (72.0)	
**Marital status**				
Married/cohabiting	122 (69.7)	142 (81.1)	264 (75.4)	0.013
Single/separated	53 (30.3)	33 (18.9)	86 (24.6)	
**Education**				
No formal/Primary	80 (45.7)	90 (51.4)	170 (48.6)	0.042
Secondary	65 (37.2)	44 (25.2)	109 (31.1)	
College	30 (17.1)	41 (23.4)	71 (20.3)	
**Employment**				
Employed	67 (38.3)	76 (43.4)	143 (40.9)	0.328
Unemployed	108 (61.7)	99 (56.6)	207 (59.1)	
**Health facility**				
Majengo	118 (67.4)	117 (66.9)	235 (67.1)	0.909
St. Joseph	57 (32.6)	58 (33.)	115 (32.9)	
**Clinical characteristics**				
**Gravidity**				
Primigravida	66 (37.7)	85 (48.5)	151 (43.2)	0.074
Multigravida	98 (56.0)	82 (46.9)	180 (51.4)	
Grand multigravida	11 (6.3)	8 (4.6)	19 (5.4)	
**Pregnancy type**				
Planned	79 (45.1)	85 (48.6)	164 (46.9)	0.520
Unplanned	96 (54.9)	90 (51.4)	186(53.1)	
**Complications in the previous pregnancy**				
No	159 (90.9)	153(87.4)	312 (89.1)	0.027
Yes	16 (9.1)	22 (12.6)	38(10.9)	
**HIV status**(*n*** = 348)**				
Negative	158(90.3)	149(86.1)	307(88.2)	0.229
Positive	17 (9.7)	24 (13.9)	41(11.8)	

### Socio-demographic characteristics of male partners who attended ANC

Sociodemographic characteristics of male partners of the pregnant women 228 male accompany their women to either of the two subsequent visits following the invitations. Male of the pregnant partners attended to the ANC with Median age 28 (24, 35) years, 87.7% were married, 61.7% were employed, 81.1% had never tests HIV for the past 12months, 97.7% prefer HIV counselling and testing, 90.7% had never attended to the ANC before the invitations. Other characteristics are shown in Table 2.

**Table 2:** Socio-demographic characteristics of male partners attended ANC at Moshi Municipal (*N* = 228)

Characteristics	Total	Control	Intervention	
	n (%)	*n* = 81	*n* = 147	*p*-value
**Age group (in years)**				
18–34	172 (74.8)	57 (69.5)	115 (77.7)	0.171
35–49	58 (25.2)	25 (30.5)	33 (22.3)	
*Median (Interquartile range)*	28 (24, 35)			
**Marital status**				
Married/cohabiting	200(87.7)	72 (88.9)	128 (87.1)	0.690
Single/separated	28 (12.3)	9 (11.1)	19 (12.9)	
**Education**				
None/Primary	99 (43.4)	25 (30.9)	74 (50.3)	0.010
Secondary	49 (21.5)	24 (29.6)	25 (17.0)	
College	80 (35.1)	32 (39.5)	48 (32.7)	
**Employment**				0.635
Employed	140 (61.7)	51 (63.8)	89 (60.5)	
Unemployed	87 (38.3)	29 (36.2)	58 (39.5)	
**Ever tested for HIV in the past year**				
No	184 (81.1)	69 (85.2)	115 (78.8)	0.237
Yes	43 (18.9)	12 (14.8)	31 (21.2)	
**Prefer HIV testing and counselling**				
No	5 (2.2)	5 (6.2)	0 (0)	0.002
Yes	222 (97.8)	76 (93.8)	146 (100.0)	
Accompanying partners*				
Never	138 (90.7)	55 (76.4)	83 (83.8)	0.223
Once/twice	33 (19.3)	17 (23.6)	16 (16.2)	
**Benefits of ANC visits**				
No benefit	11 (4.9)	3 (3.7)	8 (5.5)	< 0.001
Make partner safe	20 (8.8)	14 (17.3)	6 (4.1)	
HIV testing	82 (36.1)	17 (30.0)	65 (44.5)	
Education for us and new baby	114 (50.2)	47 (58.0)	67 (45.9)	

Key: * frequency do not tally due to missing values

### Efficacy of male invitation

**Fig. 3:**
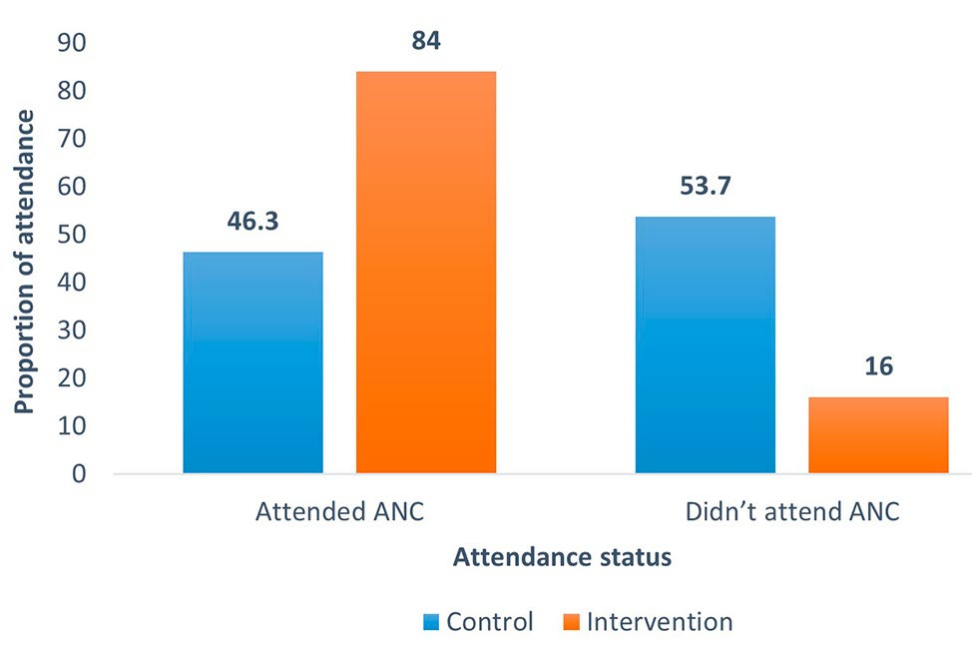
The response rate of the male partners to ANC visit after invitation

Of 350 participants, 147 and 81 pregnant women were in the intervention and control arms respectively, attendance was recorded in either of the two subsequent antenatal visits as scheduled. The response rate was 84% (147/175) for the intervention arm and 46.3% (81/175) for the control arm as represented in Fig. 3.

### Average treatment effect (ATE)

The results demonstrate a positive and statistically significant treatment effect of the mobile phone intervention on ANC attendance, as measured by a relative risk (RR) of 1.458113 (95% CI: 1.330359–1.598134, *p* < 0.001). This implies that pregnant individuals who received the mobile phone intervention, involving phone calls and engagement with health providers, were 45.8% more likely to attend ANC compared to those whose partners received the intervention. The estimated effect of 0.3771429 provides a robust indication of the intervention’s efficacy, as supported by the provided statistical measures in Table 3.

**Table 3:** Average treatment effect for mobile phone intervention on ANC attendance

	ATE (RR)	Robust std.Error	95% CI	*P*-value
Pregnant partner only				
Pregnant partner and phone call and health provider	1.458113	1.047895	(1.330359–1.598134)	< 0.001

### Factors associated with male partner ANC attendance following the invitation

In Multivariable logistic regression, marital status, education level, HIV status and gravity were the significant maternal factors that were associated with male partner attendance to ANC visit.

Pregnant women who were married/cohabiting were more likely to attend ANC visit with their partner compared to single/separated pregnant women (AOR: 6.4; 95% CI 3.26–12.56). Educated pregnant women were less likely to attend ANC visits with their partner compare to none/primary education (AOR: 0.43; 95% Cl (0.24–0.76). Women living with HIV disease were more likely to attend ANC visit with their partners compared to HIV negative pregnant women (AOR:2.56; 95% Cl (1.10–5.92). Multigravida women were less likely to attend of ANC visit with their partners compared to the primigravida women (AOR:0.17; 95% Cl (0.09–0.33) as shown Table 4.

**Table 4:** Factors associated with male partner ANC attendance following the invitation (*N* = 350)

Characteristics	Control n (%)	Intervention n (%)	cOR (95% CI)	*P*-value	aOR(95%CI)	*P*-value
**Maternal age**						
18–34	141(80.6)	154 (88.0)	1.42(0.79–2.56)	0.239	1.59(0.75–3.39)	0.226
35–49	34 (19.4)	21 (12.0)	1		1	
**Marital status**						
Single/Separated	122(69.7)	142 (81.1)	1		1	
Married/cohabiting	53 (30.3)	33 (18.9)	3.47(2.09–5.74)	< 0.001	6.40(3.26–12.56)	< 0.001
**Education mother**						
None/Primary	80 (45.7)	90 (51.4)	1		1	
Secondary	65 (37.2)	44 (25.2)	0.44(0.27–0.72)	0.001	0.43(0.24–0.76)	0.004
College	30 (17.1)	41 (23.4)	1.36(0.72–2.57)	0.342	1.37(0.62-3.00)	0.437
**Employment**						
Unemployed	67 (38.3)	76 (43.4)	1		1	
Employed	108(61.7)	99 (56.6)	1.22(0.78–1.92)	0.380	1.02(0.57–1.83)	0.951
**Pregnancy type**						
Unplanned	79 (45.1)	85 (48.6)	1		1	
Planned	96 (54.9)	90 (51.4)	1.68(1.08–2.63)	0.023	1.37(0.78–2.42)	0.276
**HIV Status**						
Negative	158(90.3)	149(86.1)	1		1	
Positive	17 (9.7)	24 (13.9)	1.73(0.82–3.66)	0.152	2.56(1.10–5.92)	0.029
**History of pregnancy complication**						
No	159(90.9)	153(87.4)	1		1	
Yes	16 (9.1)	22 (12.6)	0.99(0.48–2.01)	0.970	1.63(0.71–3.77)	0.249
**Gravidity**						
Primigravida	66 (37.7)	85 (48.5)	1		1	
Multigravida	98 (56.0)	82 (46.9)	0.42(0.26–0.67)	< 0.001	0.17(0.09–0.33)	< 0.001
Grand multigravida	11 (6.3)	8 (4.6)	1.26(0.39–4.03)	0.696	0.76(0.20–2.94)	0.691

*cOR-Crude odds ratio. *aOR-Adjusted odds ratio. *Adjusted for maternal age, Marital status, maternal education status, maternal employment status, pregnancy type, HIV status, History of pregnancy complication, and gravidity

## Discussion

This interventional study assessed the efficacy of a mobile phone intervention by a health care provider to invite male partners to attend ANC with their pregnant partners for HIV counselling and testing. According to this study, 84% of pregnant women in the intervention arm and 46.3% in the control arm returned with their male partners.

This prevalence is higher than those reported from other studies done in Southern Tanzania following official invitation 53.5% male attended [21], following written invitation 66% of male attended following written invitation in Mbeya Tanzania and Only 30% of pregnant women present for couples counselling with their partners after being invited during Prevention of Mother To Child Transmission of HIV in Tanzania [22]. Another study conducted in Malawi 72% of males attended to the ANC following mobile phone intervention [20], This high return rate in the intervention arm might be due to the influence of healthcare provider who directly communicate with male partners via phone call and text message. Not only that compared to other interventions which usually use pregnant woman to presents a leaflet or invitation card to a male partner all this interventions relay to pregnant women which might be ineffective because a pregnant woman might misplace an invitation and the message will not reach to a male partner as it was intended. On other hand the high attendance rate in intervention arm might be due to the reasons that through direct communication by the heath care provider gives a male partner opportunity to ask and have an understanding relating to the reason of why is important to come for the ANC with the pregnant partner which might be difficult explained by the pregnant woman. Futhermore the use of mobile phone since it is easy for the person to move with it from place to place any where the person can pick the phone and receive information and discuss the possibility of being around to the next visit which might be difficult to have it in the control arm where the pregnant women is the one who supposed to provide the information. This is supported by other research studies which found that if medical personnel provided the invitation, it had a greater influence than an invitation from the pregnant partner on its own [23, 24]. However other studies stipulate intimate partner violence and infidelity contribute to poor ANC attendance among male partners [13]. However, the return rate in the control arm from this study show higher rate compared to those of other studies. Example, return rate following verbal invitation was found to be 11% in Kenya [25] and 12% in Mbeya, Tanzania [26]. The higher return rate in this study can be explained as due to different geographical location of the study site. Another reason is the quality-of-care services during the ANC further influence the attendance rate. Also, Moshi municipal is among the area where most of its population are educated and are easy understand the importance of attending ANC with their partners. Other study observe the similar findings [27]. The study was done in Municipal which is in between rural and town in which a lot of its population easy access the health facilities compared to others.

On the factors associated with pregnant women return with their partners, married women are likely to turn to the clinic with their partners. This finding may be explained by married man to her partners feels more comfortable to go with to the ANC compared to unmarried. Multigravida women are less likely to turn to clinic with partners compared with prime gravida women. This finding may be explained by multigravida have experience on what is done to the ANC visits compared to prime gravida which influence prime gravida to turn with their partners as they go to gain new experience.

Although the findings from this study are comparable to other similar studies, it should be discussed in light of the following limitations. The study was conducted in one geographical location in an urban setting. It opens the way for further studies using a larger sample size and different geographical setting.

## Conclusion

A combined mobile phone intervention from a healthcare provider plus verbal invitation from the pregnant women was more effective in increasing male partners’ attendance at ANC and HIV counselling and testing. The combined approach is thus recommended for improving ANC attendance and HIV testing of male partners who do not accompany their pregnant partners to antenatal clinics during their first visits. The integrated mobile phone intervention, combining proactive outreach from healthcare providers and concurrent verbal invitations extended by pregnant women, significantly increased male partners’ attendance at antenatal care (ANC) and facilitated engagement in HIV counseling and testing. Our study advocates for the widespread adoption of this multifaceted strategy, emphasizing the strategic advantage of employing both verbal and mobile communication channels to invite male partners during initial antenatal visits.

This research contributes substantively to discussions on optimizing reproductive health interventions, highlighting the practicality and scalability of integrating mobile technology and interpersonal communication channels. The success of this approach underscores its potential to address the challenge of limited male participation in ANC, offering promise for broader implementation within diverse healthcare settings. Future studies should explore the sustained impact and mechanisms influencing intervention success, contributing to ongoing efforts to enhance male partner involvement in maternal care and improve health outcomes.

## Data Availability

The datasets used and/or analyzed during the current study are available from the corresponding author on reasonable request.

## Abbreviations

AIDS: Acquired Immunodeficiency syndrome
aOR: Adjusted Odds Ratio
ANC: Antenatal Care
CRERC: College Research Ethics Review Committee
cOR: Crude Odds Ratio
DMO: District Medical Office
HIV: Human Immunodeficiency virus
KCMUCo: Kilimanjaro Christian Medical University College
MOHCDGEC: Ministry of Health, Community Development, Gender, Elders and Children
MoHSW: Ministry of Health and Social Welfare
PMTCT: Prevention of Mother-to-Child Transmission of HIV
UNAIDS: United Nations Programme on HIV and AIDS
WHO: World Health Organization
